# Glow Tech in Adult Female Acne: Preliminary Real‐World Experience

**DOI:** 10.1111/jocd.70734

**Published:** 2026-02-09

**Authors:** Rafael Rodrigo Crisanto de Oliveira

**Affiliations:** ^1^ Centro de Ciências da Saúde, Universidade Federal da Paraíba João Pessoa State of Paraíba Brazil

Dear Editor,

1

Adult female acne (AFA) represents a global health challenge with distinct clinical features, often requiring a multifaceted management strategy due to its recalcitrant nature and significant psychosocial burden [1, 2]. In a real‐life clinical practice setting at a specialized center in Brazil, it has been observed that the integration of systemic therapy with sequential in‐office procedures may offer a pragmatic pathway for treatment. This preliminary observational case series of seven Brazilian women (ages 18–45, Fitzpatrick skin types II–IV) is reported to share clinical observations derived from a combined approach referred to as the “Glow Tech” sequence.

This work should be interpreted as a retrospective, exploratory case series reflecting real‐world clinical experience rather than confirmatory evidence. Eligible patients were women aged 18–45 years, with no spironolactone use or esthetic procedures in the preceding 6 months, and willing to discontinue concurrent acne treatments. The study was conducted in accordance with the Declaration of Helsinki, and all patients provided informed consent for the anonymized use of their data and photographs. These observations primarily reflect hormonally influenced AFA phenotypes and should not be generalized to all clinical variants of adult female acne.

All patients presented with mild‐to‐moderate inflammatory acne, predominantly in the mandibular region (U‐zone). Underlying conditions, including Polycystic Ovary Syndrome (PCOS) or endometriosis, were confirmed in all subjects via hormonal evaluation and pelvic/transvaginal ultrasound. PCOS was diagnosed according to the Rotterdam criteria, with all affected patients fulfilling at least two of the following: hyperandrogenism, ovulatory dysfunction, and polycystic ovarian morphology [1].

These patients had previously reported suboptimal results or low adherence to conventional topical monotherapies, reflecting the complexity of AFA in daily practice. The “Glow Tech” sequence represents an institutional multimodal clinical approach currently under exploratory evaluation. This series represents a subset of AFA patients with clear hormonal influence, and therefore the observations cannot be generalized to the entire spectrum of adult female acne.

The treatment consisted of a 4‐month regimen involving:

1. *Systemic support*: Spironolactone (50 mg/day) administered orally in the morning throughout the period and for 3 months following the protocol [1].
2. *In‐office procedure (monthly sessions* × *4*): A sequential procedure performed in a single visit following this strict sequence:
  - *Step 1 (carbon mask)*: Application of a thin layer of carbon paste (black peel) to the entire face [3, 4].
  - *Step 2 (laser application)*: Utilization of the *Solon Platform (LMG Lasers)* with the 1064 nm Q‐switched Nd:YAG handpiece. Parameters: Fluence of 2 J/cm^2^, Spot size of 6 mm, and Frequency of 10 Hz. The laser was applied over the carbon mask [3, 4].
  - *Step 3 (Cleansing)*: Thorough removal of carbon residue
  - *Step 4 (PDRN)*: Immediate topical application of Polydeoxyribonucleotide (PDRN) to the treated area [5].
  - *Step 5 (retinoic acid mask)*: A 10% Retinoic Acid mask (yellow peel) applied over the PDRN, removed by the patient with water after 8 h [4].
3. *Supportive homecare*: Focused on barrier repair (hyaluronic acid 5% and glycerin 1%). Adapalene 0.1% + Benzoyl Peroxide 2.5% gel was used only as a spot treatment on active lesions, suspended for 7 days post‐procedure.

Efficacy was assessed via standardized photographs evaluated by three independent board‐certified dermatologists using the Investigator's Global Assessment (IGA) and the Adult Female Acne Scoring Tool (AFAST). Statistical analysis was performed using Python (SciPy library). Statistical outputs are presented purely as descriptive indicators rather than inferential evidence.

Quantitative analysis showed a reduction in acne severity: the mean baseline IGA score of 2.38 ± 0.52 reduced to 0.95 ± 0.59, while the AFAST scale decreased from 1.38 ± 0.36 to 0.48 ± 0.18 (Figure 1). It must be emphasized that these findings are purely illustrative given the small sample size (*n* = 7), serving to document the clinical trajectory of these specific cases (Figure 2) rather than establishing a statistically powered conclusion. Patient satisfaction, recorded through a non‐validated 5‐point Likert scale, averaged 4.8. While the use of a non‐validated tool is a limitation, it provides a qualitative glimpse into patient perception. A 6‐month follow‐up demonstrated maintenance of clinical improvement in all patients.

**FIGURE 1:**
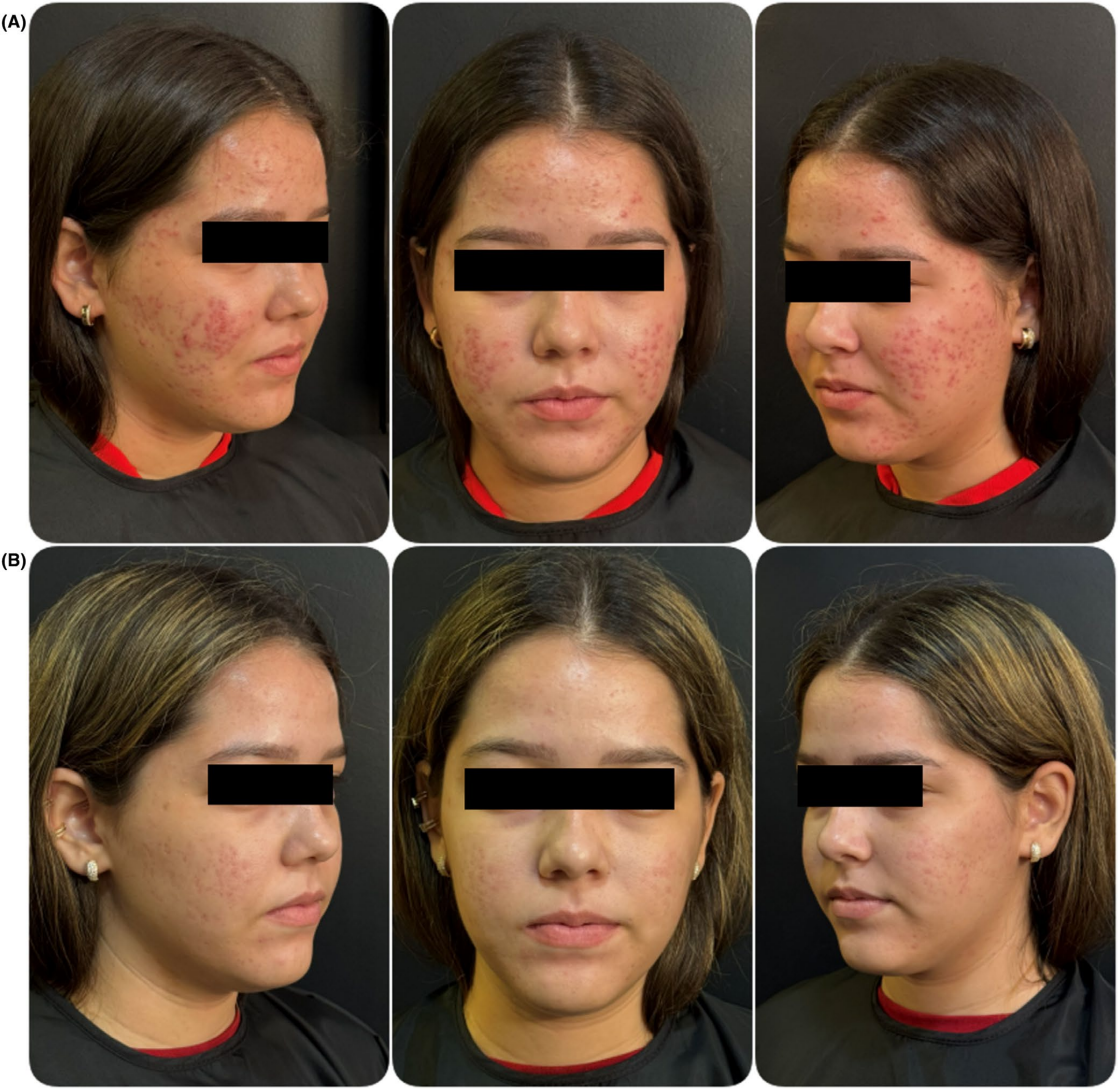
Standardized close‐up clinical photographs of a representative patient at baseline and 30 days after completing the four‐session “Glow Tech” protocol. (A) Baseline (pre‐treatment): Moderate adult female acne is observed, characterized by the presence of inflammatory papules, pustules, and scattered comedones, with a predominance in the mandibular region (U‐zone). (B) 30 days post‐treatment: A marked reduction in inflammatory lesions and comedones is evident across the treated areas. The skin demonstrates improved texture, increased radiance, and a visible decrease in post‐inflammatory hyperpigmentation. The post‐treatment illustrates a significant clinical improvement. Photographs were taken under standardized lighting and angle conditions.

**FIGURE 2:**
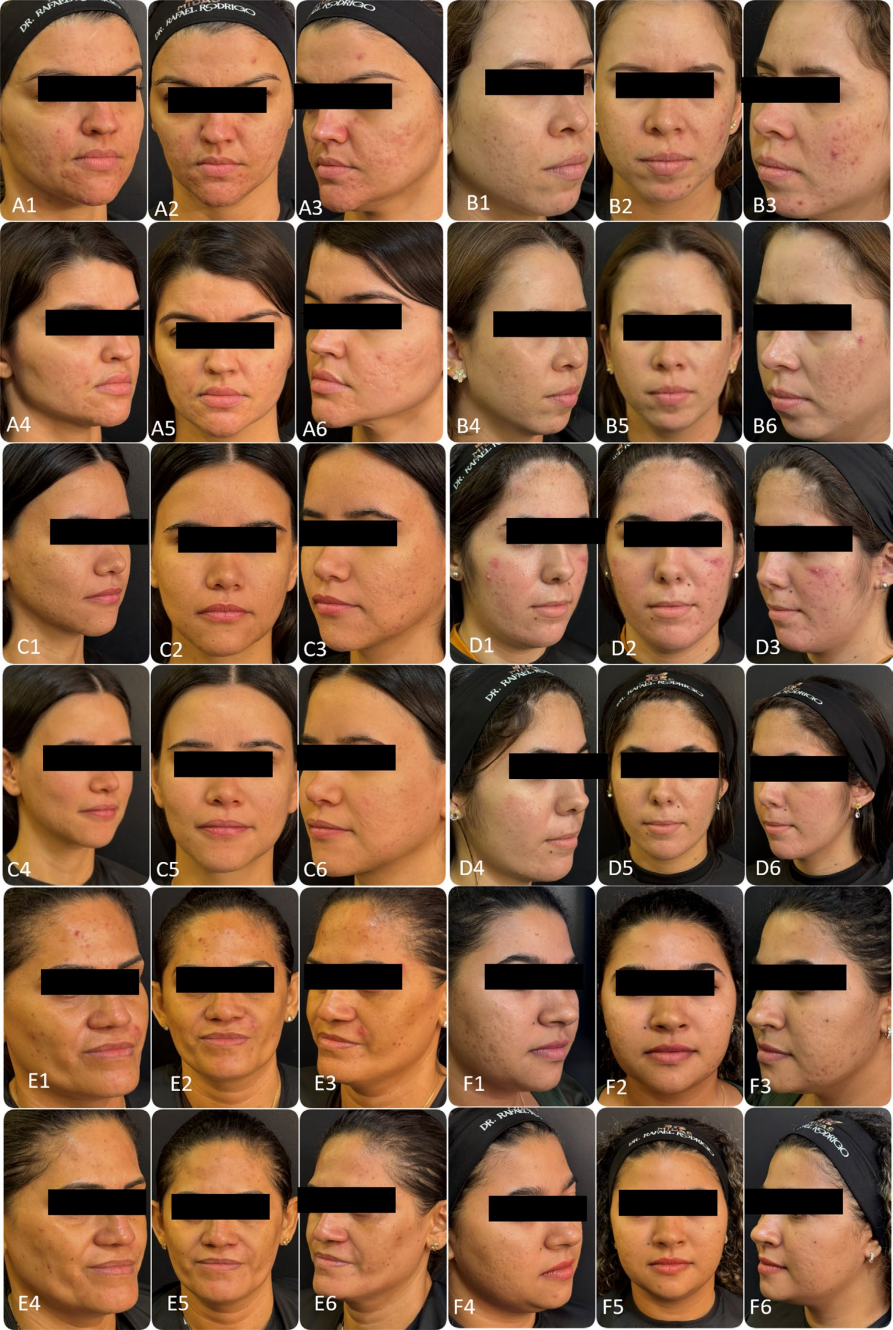
Serial clinical documentation of six patients (A–F) demonstrating the consistency of the “Glow Tech” protocol outcomes. For each patient panel, the upper row represents the baseline state (e.g., A1–A3), while the lower row shows the results 30 days after the fourth and final session (e.g., A4–A6). The composite images reveal a uniform reduction in inflammatory papules and pustules, alongside a visible improvement in overall skin quality and radiance across the cohort.

The rationale behind this sequence lies in the complementary mechanisms of the agents involved, as combined therapies often show superior efficacy compared to monotherapy [6]. While spironolactone addresses the systemic androgenetic trigger, the carbon peel with 1064 nm Nd:YAG provides a keratolytic effect and reduction of *C. acnes* load [3]. The subsequent application of PDRN—an A2A receptor agonist—may contribute to a more favorable inflammatory environment, potentially improving tolerance to the retinoic acid mask [4, 5].

Regarding safety, expected adverse effects were observed across the entire cohort. Erythema and scaling occurred in 100% of patients during the first post‐procedure week, often accompanied by transient skin sensitivity. These manifestations were expected, self‐limiting, and effectively managed with the prescribed hydration and strict photoprotection regimen. No severe adverse events or long‐term complications were recorded, suggesting the protocol is well‐tolerated within a real‐world clinical setting.

The primary goal of this report is to exemplify a treatment possibility for AFA rather than to establish a new standardized method. Significant limitations, including the small cohort and lack of a control group, preclude definitive conclusions. However, these preliminary observations may contribute to the clinical discussion on optimizing outcomes for adult women. Further large‐scale, randomized trials are essential to validate these initial clinical impressions. Rather than proposing a definitive therapeutic protocol, the “Glow Tech” sequence aims to stimulate clinical discussion regarding pragmatic, multimodal strategies in daily dermatologic practice.

## Ethics Statement

The author has nothing to report.

## Conflicts of Interest

The author declares no conflicts of interest.

## Data Availability

The data that support the findings of this study are available from the corresponding author upon reasonable request.
